# Laparoscopic cholecystectomy during the COVID-19 pandemic in a tertiary care hospital in Germany: higher rates of acute and gangrenous cholecystitis in elderly patients

**DOI:** 10.1186/s12893-022-01621-z

**Published:** 2022-05-10

**Authors:** Mirhasan Rahimli, Cora Wex, Felix Wiesmueller, Frederike Weber, Maximilian Dölling, Alexander Rose, Sara Al-Madhi, Mihailo Andric, Roland Croner, Aristotelis Perrakis

**Affiliations:** 1 Department of General, Visceral, Vascular and Transplant Surgery, University Hospital Magdeburg, Leipziger Str. 44, 39120 Magdeburg, Germany; 2 Division of Cardiothoracic Surgery, Brigham and Women’s Hospital, Harvard Medical School, 75 Francis Street, Boston, MA 02115 USA

**Keywords:** COVID-19, Pandemic, Cholecystitis, Cholecystectomy, Hepatobiliary surgery, Laparoscopic surgery

## Abstract

**Background:**

The COVID-19 pandemic caused a global health crisis in 2020. This pandemic also had a negative impact on standard procedures in general surgery. Surgeons were challenged to find the best treatment plans for patients with acute cholecystitis. The aim of this study is to investigate the impact of the COVID-19 pandemic on the outcomes of laparoscopic cholecystectomies performed in a tertiary care hospital in Germany.

**Patients and methods:**

We examined perioperative outcomes of patients who underwent laparoscopic cholecystectomy during the pandemic from March 22, 2020 (first national lockdown in Germany) to December 31, 2020. We then compared these to perioperative outcomes from the same time frame of the previous year.

**Results:**

A total of 182 patients who underwent laparoscopic cholecystectomy during the above-mentioned periods were enrolled. The pandemic group consisted of 100 and the control group of 82 patients. Subgroup analysis of elderly patients (> 65 years old) revealed significantly higher rates of acute [5 (17.9%) vs. 20 (58.8%); p = 0.001] and gangrenous cholecystitis [0 (0.0%) vs. 7 (20.6%); p = 0.013] in the “pandemic subgroup”. Furthermore, significantly more early cholecystectomies were performed in this subgroup [5 (17.9%) vs. 20 (58.8%); p = 0.001]. There were no significant differences between the groups both in the overall and subgroup analysis regarding the operation time, intraoperative blood loss, length of hospitalization, morbidity and mortality.

**Conclusion:**

Elderly patients showed particularly higher rates of acute and gangrenous cholecystitis during the pandemic. Laparoscopic cholecystectomy can be performed safely in the COVID-19 era without negative impact on perioperative results. Therefore, we would assume that laparoscopic cholecystectomy can be recommended for any patient with acute cholecystitis, including the elderly.

## Introduction

After the outbreak of pneumonia caused by Severe Acute Respiratory Syndrome Coronavirus 2 (SARS-CoV-2) at the end of 2019 in Wuhan City, Hubei Province in China, the World Health Organization (WHO) declared a global health emergency in early 2020 and named this disease COVID-19 [1]. Soon COVID-19 reached pandemic status and triggered an international health crisis. The surgical disciplines were also strongly influenced by the pandemic. Numerous elective surgeries have been postponed to create treatment capacity for COVID-19 patients. In several centers, elective gastrointestinal and bariatric operations, elective endoscopies and liver transplants have been cancelled [2].

Several surgical societies published recommendations for clinical management during the COVID-19 pandemic [3]. The European Society of Trauma and Emergency Surgery (ESTES) issued a recommendation for postponing all elective surgeries. Only patients with cancer, highly symptomatic benign disease, significant infections, patients at risk for life-threatening outcomes or harm by delay should be scheduled for surgery based on individual and multidisciplinary consideration [4].

The treatment of acute cholecystitis also introduced a challenge for physicians. Even before admitting a patient to surgery there were unsolved questions, i.e. is the procedure worth the risk of possibly exposing the patient to hospital acquired COVID infection or, vice versa, expose the hospital population to a virus coming from a surgical patient? Therapy of acute cholecystitis ranged from conservative treatment with antibiotics to surgical procedures and percutaneous cholecystostomy [3]. In Spain, 96.7% of centers cancelled elective cholecystectomies during the initial period of the pandemic. Conservative therapy was chosen over procedure in 90% of patients presenting with acute cholecystitis [5]. An Irish study showed an increased incidence of acute calculous cholecystitis during the early phase of COVID-19 pandemic [6]. A Milanese study presented an overall success rate of 87.5% after percutaneous cholecystostomy with a mean post-procedural hospitalization length of nine days. Fifty percent of these patients underwent a definitive laparoscopic cholecystectomy [7].

Nevertheless, laparoscopic cholecystectomy remains the gold standard for acute cholecystitis—even during a pandemic. Percutaneous cholecystostomy is an alternative in high-risk patients if antibiotic therapy fails [3].

The first national lockdown in Germany came into force on March 22, 2020 [8]. This meant extensive contact restrictions among the people in Germany. Like at numerous other medical centers we postponed all elective procedures, including elective cholecystectomies [2].

The aim of this study is to investigate the impact of the COVID-19 pandemic on perioperative outcomes of laparoscopic cholecystectomies with regard to acute cholecystitis at a tertiary care hospital in Germany. For this purpose, we examined laparoscopic cholecystectomies in the pandemic period between March 22, 2020 and December 31, 2020. We then compared these outcomes to patients undergoing procedure the same period of the previous, non-pandemic year.

## Patients and methods

### Patients

A total of 252 patients underwent laparoscopic cholecystectomy at the University Hospital Magdeburg between 2019 and 2020. We selected patients who underwent laparoscopic cholecystectomy in the period from March 22 to December 31 for each year, 2019 and 2020. Open cholecystectomies, cholecystectomies as part of another operation and cholecystectomies with diagnosis of gallbladder carcinoma were excluded. We also excluded patients under 18 years of age. Ten patients in “pandemic year” 2020 and 10 patients in 2019 with intraoperatively converted procedures, i.e., laparoscopy to laparotomy, were excluded from the main analysis in order to reduce bias in perioperative outcomes. We identified 182 patients who met the selection criteria. The patient cohort was divided in two groups. The operations performed in the period from March 22 to December 31 in 2020 were considered as pandemic group consisting of 100 patients. The second group served as a control group and included remaining 82 patients who underwent a laparoscopic cholecystectomy in the same period of 2019.

### Therapy concept during COVID-19 pandemic

As a tertiary care hospital, we perform laparoscopic cholecystectomy for acute, chronic or other diseases of the gallbladder. According to international recommendations and in line with our assessment of the situation in 2020, we postponed many elective surgeries during COVID-19 pandemic. Postponed procedures also included elective cholecystectomies due to chronic symptomatic gallstone disease or gallbladder cholesterol polyps. During the pandemic, patients with acute symptoms caused by acute or chronic cholecystitis first received conservative therapy with antibiotics. If antibiotic therapy failed within the first 1–3 days, patients underwent laparoscopic cholecystectomy. Patients with acute cholecystitis and choledocholithiasis underwent endoscopic retrograde cholangiopancreatography (ERCP) with stone extraction and antibiotic therapy. Early elective cholecystectomy was performed if patients still exhibited symptoms despite antibiotic therapy.

While only emergency cholecystectomies were performed in the early pandemic phase during the first national lockdown, elective cholecystectomies, including postponed cases, were also performed in the pandemic group in later weeks and months after de-escalation of the lockdown measures since May 2020.

### Definitions

We defined all postoperative complications as overall morbidity. The severity of complications were determined using the Clavien-Dindo classification [9].

The length of stay (LOS) indicates the duration of postoperative hospitalization. Cholecystectomy was defined as “early” if performed within 14 days of symptom onset. Urgent and semi-urgent cholecystectomies were performed within 72 h and 4–14 days after symptom onset, respectively [10].

### Statistical analysis

All patient data was collected retrospectively. We compared patient characteristics and perioperative parameters between the pandemic and control group. Furthermore, we performed a subgroup analysis comparing outcomes in elderly patients (> 65 years old) between pandemic and control group. The Mann–Whitney U test and Pearson’s chi-squared test or Fisher’s exact test were applied for statistical comparison of two groups depending on the type of variable. We used the mean and standard deviation (SD) or the number of cases and percentages for the data presentation in accordance with the type of data. P-values of < 0.05 were considered to be statistically significant.

All data analysis was performed with IBM SPSS Statistics for Windows, Version 26 (IBM Corp., Armonk, NY, USA).

## Results

### Patient characteristics

Patient characteristics are shown in Table 1. The proportion of female patients was higher in both the pandemic and control group and without any significant difference between the groups. The entire cohort consisted of 78 male (42.9%) and 104 female (57.1%) patients. Mean age and body mass index (BMI) were 55.2 (SD 18.2) years and 29.8 (SD 6.6) kg/m^2^ in the whole patient cohort, respectively. The distribution of elderly patients (> 65 years old) was similar in both groups. There was no significant difference between the groups in terms of age, BMI, American Society of Anesthesiologists (ASA) score and previous abdominal surgery.

**Table 1:** Patient characteristics and preoperative parameters

	Control group n (%) or mean (SD)	Pandemic group n (%) or mean (SD)	p-value	Total
Total (n)	82 (45.1)	100 (54.9)		182 (100.0)
Sex				
*Male*	32 (39.0)	46 (46.0)	0.344	78 (42.9)
*Female*	50 (61.0)	54 (54.0)		104 (57.1)
Age; years	54.4 (19.0)	55.9 (17.5)	0.606	55.2 (18.2)
Number of elderly patients; > 65 yo	28 (34.1)	34 (34.0)	0.983	62 (34.1)
BMI; kg/m^2^	29.7 (6.3)	29.9 (6.9)	0.971	29.8 (6.6)
ASA				
1	4 (4.9)	8 (8.0)	0.637	12 (6.6)
2	56 (68.3)	63 (63.0)		119 (65.4)
3	22 (26.8)	28 (28.0)		50 (27.5)
4	0 (0.0)	1 (1.0)		1 (0.5)
Previous abdominal surgery	26 (31.7)	25 (25.0)	0.316	51 (28.0)
Cholecystitis	20 (24.4)	37 (37.0)	0.068	57 (31.3)
Gangrenous cholecystitis	2 (2.4)	7 (7.0)	0.188	9 (4.9)
Biliary pancreatitis	12 (14.6)	19 (19.0)	0.436	31 (17.0)
Choledocholithiasis	27 (32.9)	26 (26.0)	0.306	53 (29.1)
ERCP with SE	18 (22.0)	18 (18.0)	0.506	36 (19.8)
ERCP with BSI	1 (1.2)	4 (4.0)	0.380	5 (2.7)

*ASA* American Society of Anesthesiologists, *BMI* body mass index, *BSI* biliary stent implantation, *ERCP* endoscopic retrograde cholangiopancreatography, *SD* standard deviation, *SE* stone extraction, *yo* years old

### Preoperative parameters

In this study, we grouped acute cholecystitis and severe chronic cholecystitis with acute symptoms, coining both simply as “cholecystitis”. Twenty patients (24.4%) in the control group and 37 patients (37.0%) in the pandemic group were diagnosed with cholecystitis requiring laparoscopic cholecystectomy (Table 1). This difference was not statistically significant (p = 0.068).

Numbers of patients diagnosed with gangrenous cholecystitis [2 (2.4%) vs. 7 (7.0%); p = 0.188] and biliary pancreatitis [12 (14.6%) vs. 19 (19.0%); p = 0.436] also did not differ significantly between both groups. There was no difference in numbers of cases diagnosed with choledocholithiasis or cases treated with preoperative ERCP with stone extraction or biliary stent implantation.

### Intra- and postoperative outcomes

Table 2 illustrates intra- and postoperative outcomes. We performed more early laparoscopic cholecystectomies in the pandemic group than in the control group [19 (23.2% vs. 36 (36.0%)]. However, this difference was not statistically significant (p = 0.061). There was no significant difference between the pandemic and control group regarding length of procedure, intraoperative blood loss and length of hospitalization. Intraoperative transfusions were not administered in neither group. Both groups did not show significant differences in terms of overall morbidity, 30-day readmission and 30-day mortality. There were ten overall complications in the control and eight in the pandemic group. Inpatient readmission within 30 days following procedure was required once in the control group and twice in the pandemic group. There was no 30-day mortality in the control group. In the pandemic group, an 84-year-old patient with high comorbidity passed away on the sixth postoperative day after laparoscopic cholecystectomy due to septic shock with multi-organ failure (Clavien-Dindo V).

**Table 2:** Intra- and postoperative outcomes

	Control group n (%) or mean (SD)	Pandemic group n (%) or mean (SD)	p-value	Total
Total (n)	82 (45.1)	100 (54.9)		182 (100.0)
Early cholecystectomy	19 (23.2)	36 (36.0)	0.061	55 (30.2)
Operation time; min	94.5 (37.0)	90.0 (40.2)	0.266	92.0 (38.8)
IBL; ml	57.5 (73.9)	79.2 (124.1)	0.740	69.4 (104.8)
LOS; days	4.2 (2.4)	4.2 (3.0)	0.337	4.2 (2.8)
Overall morbidity	10 (12.2)	8 (8.0)	0.346	18 (9.9)
Severe complications (Clavien-Dindo > II)	2 (2.4)	5 (5.0)	0.460	7 (3.8)
Surgical revision	0 (0.0)	1 (1.0)	1.000	1 (0.5)
30-day readmission	1 (1.2)	2 (2.0)	1.000	3 (1.6)
30-day mortality	0 (0.0)	1 (1.0)	1.000	1 (0.5)

*IBL* intraoperative blood loss, *LOS* length of stay, *SD* standard deviation

Postoperative complications are shown in Table 3. Non-surgical complications included allergic reaction, postoperative delirium and renal failure (Clavien-Dindo II). Wound or abdominal wall hematomas occurred in four patients. Three of the hematoma complications were treated non-surgically, e.g., using skin cooling and compression (Clavien-Dindo I). Blood transfusions were required in one patient with abdominal wall hematoma (Clavien-Dindo II). One patient developed bilioma at the gallbladder bed, which was treated with a CT-guided percutaneous drainage (Clavien-Dindo IIIa). In two patients, residual stones were detected in the common bile duct after laparoscopic cholecystectomy, which were removed via ERCP with stone extraction (Clavien-Dindo IIIb). One patient underwent ERCP with bile duct stenting due to a cystic duct stump leak (Clavien-Dindo IIIb). And another patient underwent surgical revision with biliodigestive anastomosis due to an iatrogenic bile duct injury during laparoscopic cholecystectomy (Clavien-Dindo IIIb). One patient had to be surgically revised because of a wound infection with abdominal wall abscess (Clavien-Dindo IIIb).

**Table 3:** Postoperative complications

Complication	Number	Management	Clavien-Dindo Classification
Allergic reaction	1	conservative	II
Postoperative delirium	1	conservative	II
Postoperative renal failure	1	conservative	II
Wound/abdominal wall hematoma	3	conservative, cooling/compression	I
	1	conservative, blood transfusion	II
Bile leakage	2	conservative, spontaneous regression	I
Gallbladder bed hematoma	2	conservative, blood transfusion	II
Gallbladder bed biloma	1	interventional, CT-guided percutaneous drainage	IIIa
Residual stones in bile duct	2	ERCP with SE	IIIb
Cystic duct stump leak	1	ERCP with BSI	IIIb
Iatrogenic bile duct injury	1	revision, biliodigestive anastomosis	IIIb
Wound infection with abdominal wall abscess	1	surgical wound revision	IIIb
Multi-organ failure/death	1		V

*BSI* biliary stent implantation, *CT* computed tomography, *ERCP* endoscopic retrograde cholangiopancreatography, *SE* stone extraction

### Outcomes in elderly patients

In addition, we analyzed elderly patients (> 65 years old) for differences in outcomes of both groups (Table 4). There were significantly more patients diagnosed with cholecystitis and gangrenous cholecystitis in the pandemic group compared to the control group. Seven elderly patients of the pandemic group had gangrenous cholecystitis, while no patient of the control group was affected [7 (20.6%) vs. 0 (0.0%); p = 0.013]. We performed significantly more early cholecystectomies on elderly patients of the pandemic group in comparison to the control group [5 (17.9%) vs. 20 (58.8%); p = 0.001]. There was no significant difference between the groups in terms of biliary pancreatitis, operation time, intraoperative blood loss, length of stay, or morbidity and mortality.

**Table 4:** Perioperative outcomes in elderly patients (> 65 years old)

	Control group n (%) or mean (SD)	Pandemic group n (%) or mean (SD)	p-value	Total
Total (n)	28 (45.2)	34 (54.8)		62 (100.0)
Cholecystitis	5 (17.9)	20 (58.8)	**0.001**	25 (40.3)
Gangrenous cholecystitis	0 (0.0)	7 (20.6)	**0.013**	7 (11.3)
Biliary pancreatitis	6 (21.4)	9 (26.5)	0.645	15 (24.2)
Early cholecystectomy	5 (17.9)	20 (58.8)	**0.001**	25 (40.3)
Operation time; min	103.0 (33.2)	99.6 (38.6)	0.534	101.1 (36.0)
IBL; ml	65.7 (69.3)	144.7 (167.2)	0.125	109.0 (137.2)
LOS; days	5.1 (3.7)	6.1 (4.4)	0.174	5.7 (4.1)
Overall morbidity	9 (32.1)	7 (20.6)	0.301	16 (25.8)
30-day mortality	0 (0.0)	1 (2.9)	1.000	1 (1.6)

*IBL* intraoperative blood loss, *LOS* length of stay, *SD* standard deviation

Significant values (p < 0.05) marked in bold

### Perioperative outcomes in converted cases

In the period from March 22 to December 31, there was a collective of 10 patients in the "pandemic year" 2020 and 10 patients in 2019 with an intraoperative conversion from laparoscopic procedure to open surgery. There was no significant difference between pandemic and control year regarding the conversion rate if these cases were taken into account (10/92 vs. 10/110; p = 0.673). Table 5 shows the comparative analysis of perioperative outcomes of converted cases in control and pandemic periods. There was no significant differences between the groups regarding the perioperative outcomes including postoperative complications and reason for conversion. There were five overall postoperative complications in this cohort. Postoperative wound infection occurred in two patients (Clavien-Dindo I). One patient had a subcutaneous wound hematoma, that was treated using skin cooling and compression (Clavien-Dindo I). One patient developed postoperative pneumonia (Clavien-Dindo II). And one patient underwent ERCP with bile duct stenting due to an iatrogenic bile duct injury during the cholecystectomy (Clavien-Dindo IIIb). The reasons for conversion were massive inflammatory adhesions (n = 10), dissection difficulties (n = 7), bleeding (n = 2) and ventilation difficulties (n = 1).

**Table 5:** Perioperative outcomes in converted cases

	Control group n (%) or mean (SD)	Pandemic group n (%) or mean (SD)	p value	Total
Total (n)	10 (50.0)	10 (50.0)		20 (100.0)
Sex				
*Male*	8 (80.0)	9 (90.0)	1.000	17 (85.0)
*Female*	2 (20.0)	1 (10.0)		3 (15.0)
Age; years	69.9 (9.4)	67.3 (7.3)	0.684	68.6 (8.3)
Cholecystitis	10 (100.0)	10 (100.0)		20 (100.0)
Gangrenous cholecystitis	4 (40.0)	3 (30.0)	1.000	7 (35.0)
Biliary pancreatitis	1 (10.0)	1 (10.0)	1.000	2 (10.0)
Early cholecystectomy	8 (80.0)	6 (60.0)	0.628	14 (70.0)
Operation time; min	138.9 (54.5)	152.2 (64.4)	0.912	145.6 (58.5)
IBL; ml	333.3 (510.5)	235.0 (122.6)	0.356	281.6 (354.8)
LOS; days	11.5 (9.2)	7.9 (2.5)	0.739	9.7 (6.8)
Overall morbidity	2 (20.0)	3 (30.0)	1.000	5 (25.0)
30-day mortality	0 (0.0)	0 (0.0)		0 (0.0)
*Postoperative complications*				
Wound infection (CD 1)	1 (10.0)	1 (10.0)	0.547	2 (10.0)
Wound hematoma (CD 1)	0 (0.0)	1 (10.0)		1 (5.0)
Postoperative pneumonia (CD 2)	0 (0.0)	1 (10.0)		1 (5.0)
Iatrogenic bile duct injury (CD IIIb)	1 (10.0)	0 (0.0)		1 (5.0)
Total	2 (20.0)	3 (30.0)		5 (25.0)
*Reason for conversion*				
Massive inflammatory adhesions	4 (40.0)	6 (60.0)	0.315	10 (50.0)
Dissection difficulties	3 (30.0)	4 (40.0)		7 (35.0)
Bleeding	2 (20.0)	0 (0.0)		2 (10.0)
Ventilation difficulties	1 (10.0)	0 (0.0)		1 (5.0)
Total	10 (100.0)	10 (100.0)		20 (100.0)

*CD* Clavien-Dindo, *IBL* intraoperative blood loss, *LOS* length of stay, *SD* standard deviation

## Discussion

The COVID-19 pandemic had a pronounced impact on abdominal surgery including tumor and transplant surgery [2]. Whether laparoscopic cholecystectomy for acute cholecystitis should be performed or not during the pandemic was heavily debated. In particular, deciding when to perform this procedure during the course of disease turned out to be challenging [3]. In addition to antibiotic therapy and laparoscopic cholecystectomy, some groups chose percutaneous cholecystostomy for the treatment of acute cholecystitis during the COVID-19 pandemic [7, 11]. A multicenter randomized clinical trial (CHOCOLATE trial) compared laparoscopic cholecystectomy versus percutaneous catheter drainage for acute cholecystitis in high risk patients. It showed a significantly higher rate of major complications in the percutaneous drainage group (65% vs. 12%). The mortality rate was 9% in percutaneous drainage group vs. 3% in laparoscopic cholecystectomy group (p = 0.27) [12]. Due to reported high complication rate, we opted not to offer percutaneous cholecystostomy instead of surgery.

All patients with acute cholecystitis who did not respond to antibiotic therapy within three days underwent early laparoscopic cholecystectomy at our hospital. The patient characteristics and perioperative outcomes were similar in the pandemic and control group and without significant differences. However, the rates of overall and gangrenous cholecystitis were significantly higher in the pandemic group of elderly patients (> 65 years old). While there was no patient with gangrenous cholecystitis in the elderly control group, we identified seven patients with gangrenous cholecystitis in the elderly pandemic group [0 (0%) vs. 7 (20.6%); p = 0.013]. A possible association between COVID-19 infection and gangrenous cholecystitis as a late complication has been discussed in literature [13, 14]. However, no COVID-19 infection was found in any of the gangrenous cholecystitis patients of our cohort. Moreover, significantly more early cholecystectomies were performed in the elderly pandemic group. This finding could be explained by the significantly higher proportion of cases with acute cholecystitis and the lack of timely response to antibiotic therapy in this group. We suspect that many elderly patients presented with progressed disease because they initially were afraid of contracting COVID-19 from consulting with their doctors. During the first national lockdown in Germany, following a mandatory very restricted community transmission policy, strict contact restrictions have been applied. More than two people were not allowed to gather in public and a minimum distance of 1.5 m should be maintained, except for families and people living together. Non-essential shops such as hair salons, cosmetic studios, fitness studios, etc. had to close. In compliance with the distance regulation, it was still allowed to exercise alone outside. Restaurants were only allowed to offer food delivery and pickup, otherwise they had to close. In our center, visitors and patients’ relatives were not allowed to enter the hospital. Unfortunately, we did not survey our patients and do not have evidence to support this thought. However, from our own limited, anecdotal memory we can recall how several patients shared a similar story: They would stay at home as symptoms progressed and not seek professional medical care until symptoms became unbearable. The reason for this delay in seeking care was fear of exposing themselves to COVID-19 in health care facilities. Recent studies show a remarkable decrease in emergency room visits during the pandemic [15, 16]. A visit to the doctor during the pandemic could be complicated especially for elderly patients. Circumstances such as contact restrictions for relatives, lack of mobility, failure to recognize symptoms in time, could have exacerbated the situation [15]. Taken together, these factors possibly could be the explanation for higher rates of cholecystitis in elderly patients during the pandemic.

All patients at our center underwent a SARS-CoV-2 test upon admission. In addition, the patients were asked after a standardized questionnaire about COVID-19 related symptoms such as fever, contact with COVID-19 positive patients, travel history, etc. An inpatient admission took place after the COVID-19 test result was available. COVID-19 wards were set up in our institution. Colleagues from other departments were diverted to these units. Our department was not affected by this measure. In our operating rooms, surgeons, medical and paramedical staff had to wear FFP2 masks. In addition, a current negative COVID-19 test result should be available when the patient was admitted to the operating room.

Protecting patients and healthcare professionals from COVID-19 infection is a very important aspect in the pandemic. Personal protective equipment (PPE) should be worn when treating COVID-19 positive or COVID-19 suspect patients undergoing emergency laparoscopic cholecystectomy. Since the aerosolization of COVID-19 virus particles during laparoscopy is not clearly evidence-based, it is recommended that laparoscopic cholecystectomy be preferred over the open procedure in COVID-19 positive patients [17].

There was no significant difference in postoperative outcomes including morbidity and mortality between pandemic and control group, both in the overall and in the subgroup analysis. Laparoscopic cholecystectomy seems to be a safe and feasible procedure even during the COVID-19 pandemic. It was not associated with an increase of length of procedure, length of hospital stay, intraoperative blood loss, morbidity and mortality rate. We concur with Campanile et al. that laparoscopic cholecystectomy for acute cholecystitis can be performed safely in the COVID-19 era and can be regarded as treatment of choice if antibiotic therapy fails. Especially elderly patients are at risk from COVID-19 and have higher rates of acute cholecystitis. We strongly believe that this patient group would benefit from early cholecystectomy because it may shorten the overall length of the hospital stay [3]. On the other side, significant individual factors such as local COVID-19 incidence rates, resources of surgical and intensive care wards, workload of hospital staff, established treatment algorithms and procedural knowledge at each hospital should be taken under consideration.

### Limitations

This is a retrospective, single-center study. The patient cohort was rather small. In order to reduce possible bias we examined the exact time frame of 9.5 months for both years and excluded patients that received conversion from laparoscopic to open surgery from the main analysis. However, our results show that the two groups are well comparable, without any significant differences between the main pandemic and control group in all outcomes.

## Conclusion

In this study, elderly patients showed particularly higher rates of acute and gangrenous cholecystitis during the pandemic. Laparoscopic cholecystectomy was performed safely in these patients without any negative impact on perioperative results. From a surgical perspective, we would continue to recommend the laparoscopic approach for cholecystectomy in both adult and elderly patients. In addition, all appropriate preventing measures have to be taken to ensure that patients and healthcare professionals are adequately protected against COVID-19 infection.

## Data Availability

For data protection reasons, we refrained from publishing the raw data. However, the datasets used and/or analyzed during the current study are available from the corresponding author on reasonable request.

## Abbreviations

ASA: American Society of Anesthesiologists
BMI: Body mass index
BSI: Biliary stent implantation
CT: Computed tomography
ERCP: Endoscopic retrograde cholangiopancreatography
IBL: Intraoperative blood loss
LOS: Length of stay
SD: Standard deviation
SE: Stone extraction
